# Telemedicine Gatekeeping Over 15,000 Patients from Specialist Consultation Waiting Lists: A Cost-Minimization Study

**DOI:** 10.1177/26924366251388854

**Published:** 2025-10-24

**Authors:** Juliana Nunes Pfeil, Rudi Roman, Dimitris Rucks Varvaki Rados, Roberto Nunes Umpierre, Matheus Grassi de Oliveira, Ana Paula Beck da Silva Etges, Rodolfo Souza da Silva, Natan Katz, Rita Mattiello, Erno Harzheim

**Affiliations:** ^1^ School of Medicine, Federal University of Rio Grande do Sul (UFRGS), Porto Alegre-RS, Brazil.; ^2^ TelessaúdeRS, Porto Alegre, Brazil.; ^3^ Hospital de Clínicas de Porto Alegre (HCPA), Porto Alegre, Brazil.; ^4^ National Institute of Science and Technology for Health Technology Assessment (IATS), Porto Alegre, Brazil.

**Keywords:** economic evaluation, health care costs, primary health care, telehealth, telemedicine, transitional care

## Abstract

**Objective:**

This study assesses the cost-minimization of the RegulaSUS telemedicine program, reducing the necessity for face-to-face specialist consultations.

**Methods:**

A cost analysis study, utilizing a retrospective cohort design, was conducted from January 2017 to December 2019. The gatekeeping process for waiting lists for specialized consultations was performed by two groups: the RegulaSUS program through telemedicine intervention and those regulated by the usual procedures of the Ambulatory Regulation Center of the State of Rio Grande do Sul (the contemporaneous controls group). The primary outcome was to evaluate the financial impact of the RegulaSUS program on the health system costs using the Time-Driven Activity-Based Costing (TDABC) method.

**Results:**

The RegulaSUS program substantially reduced health care and societal expenditures, with gatekeeping by telemedicine costing $85.02 versus $214.45 for usual procedures—a 60% reduction. This intervention resulted in societal savings of R$7,833,882.56 over 36 months by successfully managing 15,064 patients within primary care settings, rather than referring them to specialists.

**Conclusions:**

The RegulaSUS program demonstrates a remarkable economic impact by integrating evidence-based gatekeeping protocols with telemedicine infrastructure, effectively decreasing specialist demand. This intervention provides compelling evidence for the potential of telemedicine to optimize health care resource allocation, enhance care coordination, and improve system efficiency. The outcomes present a scalable, financially sustainable model for health system strengthening that merits consideration for broader implementation across Brazil’s public health network and adaptation to comparable global health care contexts facing similar resource constraints.

## Introduction 

Health care systems worldwide face the complex challenge of balancing increasing service demands against finite resources, a universal dilemma that transcends national boundaries and health care structures.^1–4^ Extended waiting times for consultations, diagnostics, and interventions create barriers to care access and contribute to escalating health care expenditures. Over the past two decades, global health care spending has consistently risen as a percentage of gross domestic product, with financial inaccessibility to health services emerging as a pressing international concern.^1,3–5^

This global context demands innovative solutions for optimizing the allocation of health care resources^6,7^ An efficient system must strategically eliminate ineffective interventions, target effective treatments to the appropriate patient populations, and deliver services to the highest quality standards. Such optimization aligns with the contemporary value agenda in health care, which involves maximizing patient-centered outcomes while achieving a greater impact for each unit of cost.^7,8^

Robust primary health care (PHC) represents the cornerstone of effective health systems globally.^9,10^ PHC providers should manage most health conditions through continuous, coordinated services, with specialist referrals reserved for complex or uncommon presentations. Evidence consistently demonstrates that health systems with strong primary care foundations achieve superior population health outcomes, resulting in reduced unnecessary hospitalizations and more equitable health resources.^8,11^

The Brazilian health care sector presents opportunities for significant efficiency gains, which could lead to substantial economic savings.^12^ The fragmentation of the public health system undermines the possibility of achieving economies of scale in service provision. Moreover, the lack of integrated health systems and inadequate incentives for selecting cost-effective treatments are significant inefficiencies. The contributors to the current inefficiencies. In addition to the direct financial costs, inefficiencies lead to limited access to health care. In Brazil, particularly within the public health system, there is a significant patient waiting time for services.^13^

Telemedicine has been proposed as a potential tool to improve primary care by balancing access, quality, and cost of specialized medicine. Within Brazil’s health care system, primary care physicians serve as the initial gatekeepers, addressing most population health needs and referring only those cases that require specialized expertise through a structured evaluation process.^14^ In 2013, the RegulaSUS program was introduced in Brazil to address prolonged specialist appointment wait times by leveraging telemedicine innovations. It is one of several initiatives of a university-based telehealth program called TelessaúdeRS.^15,16^ A previous study examining the RegulaSUS program’s association and the proportion of management in PHC showed that ∼10.6% of individuals referred for specialized medical consultation were managed in PHC after implementing referral protocols and remote medical consultations for two years. By rigorously evaluating the cost-minimization impact of the RegulaSUS telemedicine program, this study provides economic evidence regarding the RegulaSUS telemedicine program in the gatekeeping specialist consultation list.^13^

## Materials and Methods

### Study design

This research employed a cost-analysis study design to evaluate the economic impact of telemedicine interventions through the RegulaSUS (Gatekeeping system for specialist consultations of the Brazilian Unified Health System (SUS), the TelessaúdeRS gatekeeping system, with a retrospective cohort study with contemporaneous controls.

### Setting

The Brazilian Unified Health System (SUS) is a national, universal, public, and free-of-charge health care system, funded by the government, available to all citizens. Alongside the SUS, there is a supplementary private health care sector, typically accessed through private insurance plans or out-of-pocket payments. The organizations included in our analysis operate within the public SUS framework, where TelessaudeRS acts as a supporting service. This ensures that the telemedicine interventions studied are accessible and free to patients within the public system.

Patient-specific consultation list data were obtained from the State Department of Health referral database, GERCON, from January 2017 to December 2019. The selection of the 2017–2019 timeframe was deliberate, as it represents a fully operational and stable phase of the TelessaúdeRS gatekeeping system. Specifically, this period commenced after the official adoption of the Gercon platform as the statewide referral information system (2017) and concluded before the widespread disruptions precipitated by the COVID-19 pandemic (2020). The COVID-19 pandemic, from 2020 onwards, profoundly disrupted access to specialized care throughout Brazil, leading to widespread suspension or reduction of consultations, a significant decrease in new referral requests, and a protracted and incomplete recovery of health services. Incorporating these external factors would have introduced substantial bias into any longitudinal comparisons with the pre-pandemic period.

### Participants

The study population consisted of individuals referred from PHC to specialized consultation via the GERCON platform.

### Exposure

The regulation process was done by two groups: those regulated by RegulaSUS (the TelessaúdeRS gatekeeping program) and those regulated by the usual procedures of the Ambulatory Regulation Center of the State of Rio Grande do Sul (RS) (control group).

The RegulaSUS methodology, fully detailed in Pfeil et al.,^13^ operates through a three-step process. Initially, patient waiting lists for specialist consultations undergo evaluation using regulatory protocols. Next, cases meeting the in-person consultation criteria receive authorization and are assigned a priority classification (high, medium, or low), with appointments scheduled based on availability. Finally, referrals that lack sufficient clinical information or are potentially resolvable at the primary care level should proceed to synchronous teleconsultation with the RegulaSUS team. During these sessions, specialist physicians provide diagnostic guidance or treatment recommendations to primary care doctors. When primary care physicians agree to manage patients within their practice, those referrals are removed from the waiting list.

### Usual gatekeeping process

The Ambulatory Regulation Center of Rio Grande do Sul processes specialist appointment requests chronologically, prioritizing patients based on their submission date rather than clinical urgency. While regulatory physicians evaluate referrals, they operate without a systematic literature review or evidence-based protocols. This conventional pathway examines cases individually but lacks mechanisms for comprehensive queue analysis or optimization. Although regulators may occasionally reference RegulaSUS-developed specialty referral criteria, the traditional system lacks infrastructure for direct teleconsultation between primary care physicians and specialists—a critical distinction from the gatekeeping telemedicine process.

### Variables definitions

Face-to-face consultation: In-person consultation with a specialist.

Waiting for a specialist consultation: The duration of patients’ waiting time for a consultation with a specialist.

Referrals were removed from the waiting list: The removal of patients from the waiting list occurred due to a canceled referral, patient death, successful consultation with a RegulaSUS specialist, or management within PHC.

### Outcomes

The primary outcome measured the cost analysis of teleconsultations within the regulated gatekeeping RegulaSUS process, focusing on reducing unnecessary specialist consultations. This reduction was evaluated by the proportion of patients managed within PHC following the regulation process without requiring specialist care.

The RegulaSUS group in this study removes referrals from waiting lists following successful patient management in primary care after a teleconsultation. Unfortunately, the data retrieved from the health system cannot isolate and identify specific reasons for each patient’s removal from the waiting list. Consequently, the difference in the number of referrals excluded between the two groups was attributed to case management within PHC without specialist intervention. The costs presented in our study were adjusted to reflect the 2025 levels.

A cost analysis was performed using the Time-Driven Activity-Based Costing (TDABC) method, which directly relates resource costs to the product, in this case, the RegulaSUS telemedicine program, by utilizing time, and the values are in dollars. The cost minimization of the RegulaSUS program was calculated based on the average savings generated by each referral removed from the waiting list, as well as the number of specialty consultations replaced by primary care consultations with remote consultations.

### Microcosting with TDABC

Process mapping involves determining the people, materials, and time required to complete each task within the system’s activities. It begins with selecting the structure and teams of the entire TelessaudeRS facility.

**FIG. 1.**
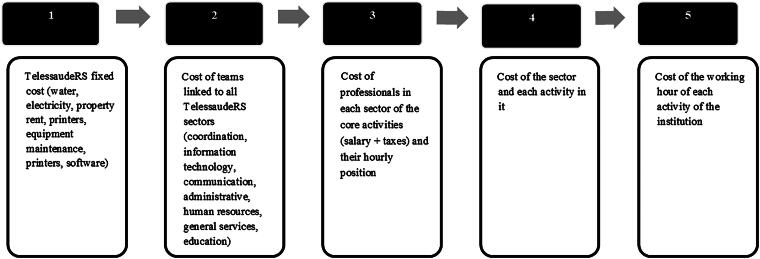
Framework for micro-costing with TDABC: adapted from Etges et al.^17^

Then, the costs of all the sectors involved in the RegulaSUS activities were estimated, identifying the share of overhead costs (light, rent, computers, printers, etc.), indirect variable costs (coordination, general services, statistics, administration, education, and communication), and direct costs of professionals working directly on the RegulaSUS, using information from the Cost Department (Fig. 1).

The resources were calculated based on the availability of professionals’ workload and the resources and structure consumed. To estimate the time required for each RegulaSUS activity, we observed and counted professionals analyzing the queue, classifying cases, and consulting with the patient’s assistant physician. The goal was to calculate the cost of all activities of the RegulaSUS telemedicine program.

For the cost analysis, the following costs were also considered:

The estimated cost of medical personnel per hour included professional compensation and the necessary infrastructure for operations.

Teleconsultations between gatekeeping and primary care physicians require an average amount of time. Length of time for state gatekeepers was assumed to be equivalent to that of TelessaúdeRS regulators.

PHC consultations were valued per visit in 2019, adjusted via the IPCA (Índice de Preços ao Consumidor Amplo, which is Brazil’s official benchmark inflation index, and reflects changes in the cost of living) from the 2023 value. Specialist consultations at public hospitals cost per visit in 2019. The standard care pathway assumed two PHC consultations per case (initial assessment and follow-up after specialist recommendation). The specialist referral pathway typically requires two consultations: an initial evaluation with test ordering and a follow-up consultation for discharge.

### Patient-related indirect costs

The daily wage loss for patients was calculated to be $28.64, based on data from the Brazilian Institute of Geography and Statistics (IBGE).

Meal expenses during travel to appointments were estimated daily.

Transportation costs varied by municipality.

### Assessing the proportion of management in PHC

We determined the proportion of management in PHC by analyzing consultations that occurred in primary care, where remote consultation was used instead of face-to-face specialist consultation in the RegulaSUS.

The cost of an unnecessary referral was measured by the cost of two consultations (a consultation for initial assessment and exams request and a second consultation to evaluate exam results, treatment follow-up, and discharge) plus the cost of travel between the requesting city and the capital, Porto Alegre, and the cost of one day off work.

We collected cost data from multiple sources:

TelessaúdeRS operational records provided gatekeeping metrics and performance indicators.

The Gercon system supplied conventional regulation processing times and case throughput statistics.

The Municipal Health Department of Porto Alegre’s Economy Center furnished primary care consultation costs and resource utilization patterns.

The public hospital complex in Porto Alegre delivered specialist consultation costs and outpatient service expenditures.

The IBGE provided wage estimation data and employment metrics for calculating productivity losses.

Commercial surveys yielded meal cost estimations based on regional market analyses.

Public transportation agencies offered travel cost calculations and route pricing between municipalities and the capital.

Sample size.

The study included all patients who met the eligibility criteria.

## Results

The estimated cost of the medical hour worked on RegulaSUS (the TelessaúdeRS gatekeeping program) was $68.22.

The average time to analyze a case in the Gercon system was 3 min (0.05 h). The average time to discuss a clinical case between the TelessaúdeRS regulator and the patient’s primary care physician was 15 min (0.25 h). A consultation at the PHC costs an average of $23.81 in 2023, and the estimated value for a consultation in primary care in 2019 was $23.75. A specialist consultation costs $46.90 in 2019.

The cost of a working day in 2019 was arbitrarily set at $28.64. The meal cost in Porto Alegre was arbitrarily set at $7.95. The displacement was for each referral to a specialized medical appointment, ranging from $1.45 to $68.61. The average was $10.15.

Considering direct costs (consultation cost) and indirect costs (travel, food, and working day), a face-to-face specialist consultation cost was $214.45 (Table 1). A primary care consultation using remote consultation instead of a face-to-face specialist consultation was $85.02 (Table 2).

**Table 1.** Specialist Consultation Time, Cost per Unit, and Gatekeeping by Service

	Time required for the task (unit)	Cost	Cost per gatekeeping
Gatekeeping by RegulaSUS	0.05 h	$3,41	$3,41
Consultation in primary care	—	$23,75	$23,75
Face-to-face consultation	—	$46,90	$93,80
Displacement	—	$10,16	$20,32
Food cost	—	$7,95	$15,91
Working day cost	—	$28,64	$57,27
Total			$214,46

**Table 2.** Regular Consultation Time, Cost per Unit, and Gatekeeping by Service

	Time required for the task (unit)	Cost per unit	Cost per gatekeeping
Gatekeeping by RegulaSUS	0.05 h	$3,41	$3,41
Tele consultations	0.25 h	$17,06	$34,11
Consultation in primary care	—	$23,75	$47,49
Total	—	—	$85,02

**FIG. 2.**
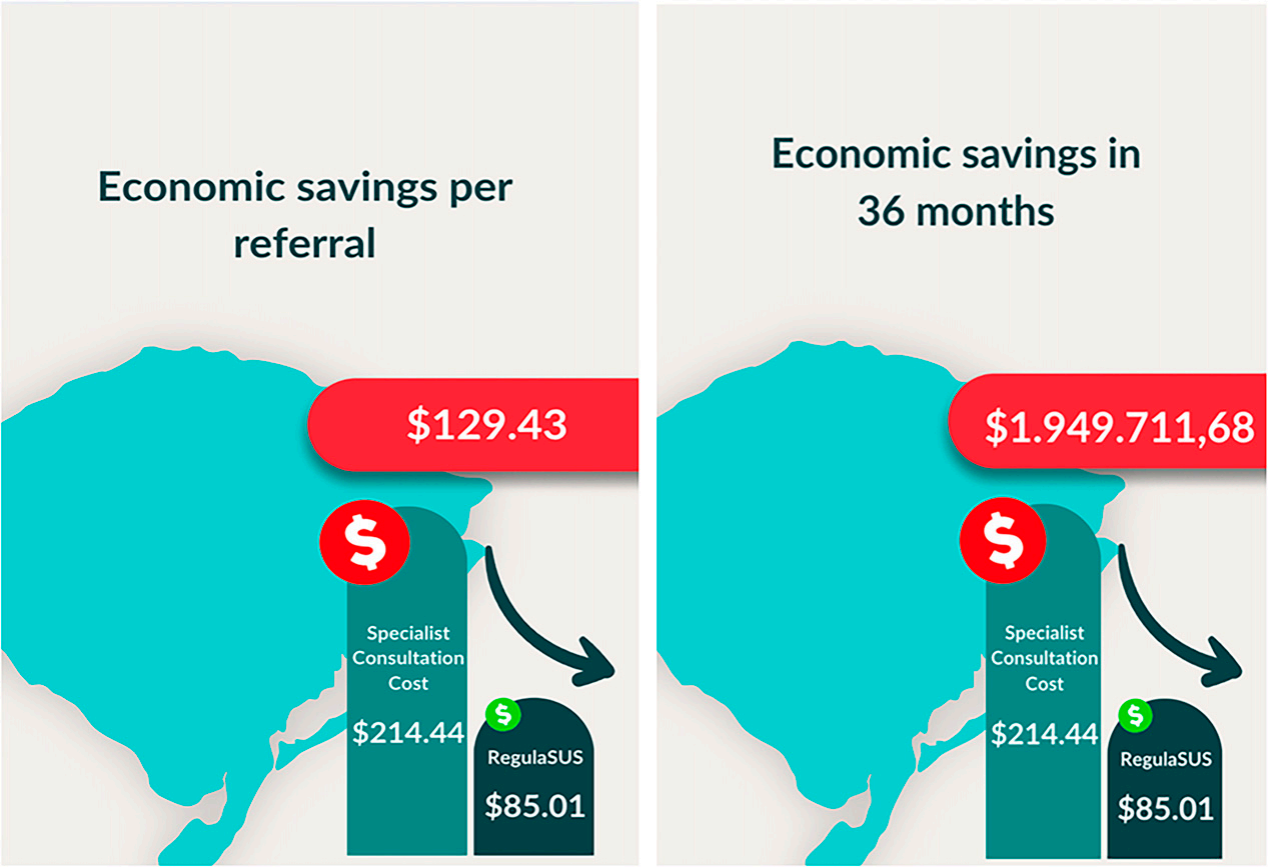
Economic Savings.

The cost-minimization analysis of RegulaSUS, considering the 15,064 referrals removed from the waiting list and treated in primary care, suggests a total economic savings to society of $1,949,760.00 over the 36 months analyzed in this study. For each individual who receives clinical management in a primary care facility following a TelessaúdeRS remote consultation, there is a savings of $129.43 for society (Fig. 2).

Considering only the direct costs to the SUS (excluding transportation, travel, food, and the patient’s working day), each referral removed from the waiting list generates a savings of $35.94, totaling $541,369.51 in savings over the 3 years analyzed in this study.

## Discussion

RegulaSUS, the TelessaúdeRS gatekeeping program, substantially reduces health care expenditures by allocating resources efficiently and optimizing care pathways through telemedicine. During the 36-month study period, the intervention saved society over $7.8 million by successfully managing 15,064 patients within primary care settings. These results confirm the effectiveness of telemedicine as a powerful cost-containment strategy that simultaneously maintains high-quality care standards.

RegulaSUS delivers comprehensive patient benefits beyond financial savings by enabling timely primary care interventions that prevent clinical deterioration while eliminating lengthy travel burdens, particularly benefiting elderly, disabled, and chronically ill populations. The program simultaneously strengthens primary care’s gatekeeping function by equipping physicians with evidence-based protocols and specialist teleconsultation support, empowering them to manage a broader spectrum of conditions locally. This proximity-based care model enhances treatment continuity, improves adherence, promotes comprehensive coordination, and yields environmental benefits through reduced patient transportation, contributing to sustainability goals while supporting the established evidence that robust primary care systems achieve superior health outcomes and more equitable health distribution. Additionally, by removing a large number of patients from the waiting list for specialized consultations, the specialized care environment itself becomes more efficient, with patients better “filtered” to benefit clinically from their encounter with the specialist doctor.^18^

Our findings align with emerging evidence on the role of telemedicine in mitigating health care access barriers. As the Organization for Economic Co-operation and Development literature highlights, long waiting times in universal, tax-funded, single-payer health systems create significant obstacles in patient care pathways, compromising equal access and potentially worsening health outcomes. While various policy interventions exist to address this challenge, our RegulaSUS program exemplifies one of the most frequently implemented primary strategies identified in a recent systematic review.^18^

The most effective way to deliver high-quality health care is to eliminate ineffective interventions, offer targeted interventions to the population most likely to benefit from them, and provide services of the highest possible quality.^1–4,19^ Organizing a health care system based on value for the patient enables the analysis of interventions over time, thereby optimizing resources and reducing errors and unnecessary or ineffective interventions.^20–22^

A previous RegulaSUS study demonstrated that this is a strategic approach to optimize waiting lists and reduce waiting times for specialized medical consultations.^23^ Our findings confirm that the project reduces costs. These results are consistent with a cost-benefit analysis already conducted for the TeleOftalmo project,^24^ which explored the economic benefits of telehealth services and the application of TDABC in health care settings, and now demonstrates the benefits of regulation for all specialties.

The RegulaSUS initiative is consistent with the principles of value-based health care by prioritizing clinical needs and attempting to manage cases within PHC settings whenever possible. The application of TDABC highlights the method’s capability to increase transparency and accuracy in cost assessment,^22^ which is critical for the transition from fee-for-service to value-based care systems. This transition is key in the context of RegulaSUS, which aims to optimize the regulation of waiting lists for specialized consultations.

Several limitations constrain our findings and warrant acknowledgment. When precise data were unavailable, we made necessary assumptions about time expenditure and regional cost variations, which could affect our estimates. The lack of transparent information regarding state gatekeeping activities forced us to extrapolate costs that may not perfectly reflect actual expenditures. The Gercon system’s limited documentation regarding referral removal reasons also introduced analytical uncertainty that could impact our conclusions. Our analysis likely underestimates the true cost differential between gatekeeping and conventional care pathways, as we excluded costs for diagnostic examinations requested at higher-complexity institutions—expenses that would further widen the cost gap between groups. This study prioritized economic outcomes while necessarily limiting its scope regarding quality metrics. Subsequent investigations should evaluate clinical outcomes, patient satisfaction, provider experience, and quality indicators to understand the impact of telemedicine.

The program’s core elements—evidence-based protocols, specialist teleconsultation, and primary care strengthening—offer adaptable solutions for health care systems worldwide struggling with resource constraints and access inequities. The RegulaSUS model creates a compelling blueprint for health care transformation applicable across diverse global contexts facing similar systemic challenges, including waiting times, fragmentation, and resource limitations. It provides robust economic evidence that telemedicine gatekeeping interventions can simultaneously improve access while containing costs.

## Conclusions

The RegulaSUS program has proven to be highly cost-effective by combining evidence-based gatekeeping protocols with telemedicine infrastructure, significantly reducing the demand for specialists. The findings strongly indicate that telemedicine can enhance health care resource allocation, improve care coordination, and increase system efficiency. This intervention offers a scalable and financially sustainable model for strengthening health systems. It deserves consideration for widespread implementation within Brazil’s public health network and adaptation in similar global health care settings facing resource limitations.

## Abbreviations Used

IBGE: Brazilian Institute of Geography and Statistics
IPCA: Índice de Preços ao Consumidor Amplo (Brazil’s official benchmark inflation index)
PHC: Primary Health Care
RegulaSUS: Gatekeeping system for specialist consultations of the Brazilian Unified Health System (SUS), the TelessaúdeRS gatekeeping system
RS: Rio Grande do Sul
SUS: Brazilian Unified Health System
TDABC: Time-Driven Activity-Based Costing
TelessaúdeRS: TelessaúdeRS (A telehealth program)
WHO: World Health Organization
